# Sero-Monitoring of Horses Demonstrates the Equivac^®^ HeV Hendra Virus Vaccine to Be Highly Effective in Inducing Neutralising Antibody Titres

**DOI:** 10.3390/vaccines9070731

**Published:** 2021-07-02

**Authors:** Kim Halpin, Kerryne Graham, Peter A. Durr

**Affiliations:** Australian Centre for Disease Preparedness (ACDP), Commonwealth Scientific and Industrial Research Organisation (CSIRO), 5 Portarlington Road, East Geelong, VIC 3219, Australia; kerryne.graham@csiro.au (K.G.); peter.durr@csiro.au (P.A.D.)

**Keywords:** Hendra virus, Hendra virus vaccine, One Health, zoonoses

## Abstract

Hendra virus (HeV) is a high consequence zoonotic pathogen found in Australia. The HeV vaccine was developed for use in horses and provides a One Health solution to the prevention of human disease. By protecting horses from infection, the vaccine indirectly protects humans as well, as horses are the only known source of infection for humans. The sub-unit-based vaccine, containing recombinant HeV soluble G (sG) glycoprotein, was released by Pfizer Animal Health (now Zoetis) for use in Australia at the end of 2012. The purpose of this study was to collate post-vaccination serum neutralising antibody titres as a way of assessing how the vaccine has been performing in the field. Serum neutralization tests (SNTs) were performed on serum samples from vaccinated horses submitted to the laboratory by veterinarians. The SNT results have been analysed, together with age, dates of vaccinations, date of sampling and location. Results from 332 horses formed the data set. Provided horses received at least three vaccinations (consisting of two doses 3–6 weeks apart, and a third dose six months later), horses had high neutralising titres (median titre for three or more vaccinations was 2048), and none tested negative.

## 1. Introduction

Hendra virus is a zoonotic, high consequence pathogen for which the natural reservoir is the four species of flying fox (*Pteropus* bat species) found on mainland Australia and the primary spillover species is the horse, from which humans are secondarily infected [1]. The virus emerged in Australia in 1994 in a racing stable in the Brisbane suburb of Hendra, where a number of thoroughbred horses developed a mysterious illness. Thirteen horses died along with their noted trainer, Vic Rail. Another man, a strapper for Vic Rail, became infected but survived [2]. Five further outbreaks occurred between 1994 and 2004, and there have been annual spillover events consistently since 2006, with most events occurring in the cooler months. Most of these have involved single horses infected in an outdoor grazing environment. Four outbreaks have involved 3 horses each, and 5 outbreaks involved 2 horses each. Two outbreaks where >3 horses were involved have each occurred in a stable setting: one being the original case at a horse training facility and one at a veterinary clinic [2,3]. The spread of the virus between the stabled horses was a result of close contact and assisted mechanical transmission of the virus [4]. Humans have also become infected because of very close contact with infected horses [3]. In total, 7 humans have become infected; the horse trainer and his strapper, three veterinarians, one veterinary nurse, and the husband of a veterinarian who assisted with a horse necropsy; of these, 4 deaths occurred [3,5]. In 2011 alone there were 18 spillover events with 24 horses dying. This cluster of events was concerning, particularly as each event represented a major threat to the humans caring for the horses. Fortunately, no humans became infected in 2011; however, the events of the year provided momentum for investment to produce a vaccine for horses which would also provide indirect protection for humans as well.

A ferret model was used to assess the immunogenicity and protective efficacy of an experimental subunit vaccine based on a recombinant soluble version of the HeV attachment glycoprotein G (HeV sG) which had shown promising results in previous studies [6,7]. Doses of 100 μg or 20 μg of HeV sG vaccine were shown to completely prevent a productive HeV infection in ferrets and provided confidence that this vaccine would work in horses as well [8]. A subunit vaccine containing recombinant HeV sG glycoprotein was then formulated in a proprietary adjuvant (Pfizer Animal Health, now Zoetis) [9]. In a series of vaccine efficacy studies, 10 horses were immunized with HeV sG and then exposed to an otherwise lethal dose of HeV by the oronasal route [9]. Vaccinated horses remained clinically healthy during the observation period after exposure to HeV. The HeV horse vaccine (Equivac^®^ HeV Hendra Virus Vaccine for Horses, Zoetis Australia Pty Ltd., Rhodes, NSW, Australia) was launched in November 2012, the first vaccine against a Biosafety Level-4 (BSL-4) agent to be licensed and commercially deployed [10].

The vaccine was initially released under a minor use permit pending registration with the Australian Pesticides and Veterinary Medicines Authority (APVMA), the government body responsible for the regulation of all agricultural and veterinary chemicals in Australia [11]. The permit enabled the vaccine to be used under strict conditions, including administration by a trained and accredited veterinary surgeon to horses that needed to be uniquely identified by a microchip, the details of which were to be also recorded on a national online registry. Several amendments have subsequently been made to the product labelling following the initial release, including six-monthly boosters and approval for use in pregnant mares. In August 2015, the vaccine was officially registered, with 12 monthly boosters approved in May 2016 [12].

The 1 mL vaccine contains not less than 100 μg of HeV sG glycoprotein antigen adjuvanted with 250 μg/dose of an immuno-stimulating complex and is administered by intramuscular injection [13]. The vaccination schedule involves a primary course of two doses 3 to 6 weeks apart, commencing as early as 4 months of age. A 3rd dose 6 months after the second dose is required, with further boosters to be administered every 12 months after the 3rd dose. All doses must be entered into an online registry at https://www.zoetis.com.au/vets-australia/ (accessed 30 March 2021) within two days of administration. All horses must be microchipped prior to administration of the first vaccine dose, and the microchip identifier also needs to be recorded in the online registry. Zoetis advises that foals born to vaccinated mares should commence vaccination at 6 months of age, and pregnant mares are not to be vaccinated during the first 45 days after conception or the two weeks prior to the expected foaling date. Since the vaccine’s release on 1 November 2012, Zoetis has sold over 750,000 doses. Zoetis has supplied details regarding sales from 2016–2019 where it sold 317,030 doses, representing 57,645 horses receiving their first dose (Supplementary Material Table S1). This is low compared to the vaccine for tetanus where approximately twice as many doses are sold per year (R. L’Estrange pers. com). A spike in Equivac^®^ HeV Hendra virus vaccine sales in 2019 can be attributed to the identification of a Hendra virus infected horse in Scone in the state of New South Wales which expanded the distribution of Hendra virus cases southwards. Estimating the percentage of horses in Australia receiving at least one dose of the vaccine in one year, based on horse population estimates [14] can be approximated (Supplementary Material Table S2). If we assume most vaccine sales are in the HeV endemic states, vaccination coverage between years 2016 and 2019 ranged from 10.1% to 13%, which at a national level decreases to 6.9–8.9%.

The serological response to the vaccine is measured using a serum neutralisation test (SNT), also known as a virus neutralisation test (VNT). Serial dilutions of serum are mixed with a standard amount of virus, incubated, and then placed onto cells. If there are antibodies in the serum, these neutralise the virus and it will not grow. An end point antibody titre can be measured in this test. Titres can range from 2 to >16,000, with the greater the amount of antibody in the serum, the higher the titre. Some horse owners opt to have their horse’s post-vaccination titre assessed, which is more expensive than the vaccination but provides some information around how their horse has responded to previous vaccinations [15]. The only laboratory in Australia which can conduct this test is the Australian Centre for Disease Preparedness (ACDP). Since the vaccine was launched, the laboratory has conducted hundreds of HeV antibody titre tests on vaccinated horses.

The Equivac^®^ HeV Hendra virus vaccine for horses has been in use for more than 7 years. We collated post-vaccine serum neutralising antibody titres as a way of assessing how the vaccine has been performing in the field. The design of the study was a retrospective exploratory data analysis using a data set which includes the age of the horse, dates of vaccinations, date of sampling and location as well as the post-vaccination SNT titre.

## 2. Materials and Methods

### 2.1. Study Population

This study consisted of equine serum samples submitted to ACDP (formerly Australian Animal Health Laboratory, AAHL) during 2014–2020 by veterinarians requesting the HeV SNT to ascertain neutralising antibody titre following a vaccination. Only samples collected from healthy microchipped horses were included in this study. The ACDP sample submission form requests relevant details including the age, sex, and breed of the vaccinated horse as well as its location, date of sampling, and the date(s) of vaccination(s).

Vaccination history was confirmed by accessing the Zoetis Hendra Vaccination Registry. The database holds historical listings on an individual horse vaccination status, including the dates when the vaccinations were administered. The Hendra Vaccination Registry is a tool designed to help vets and the public easily look up the Hendra vaccination status of horses across Australia. The information contained in this registry comprises information provided by veterinarians accredited to administer the Hendra vaccine. It is the responsibility of accredited veterinarians to enter data accurately. Zoetis does not undertake independent checks of the data entry, and therefore they cannot guarantee its accuracy. Therefore, where possible, vaccination data were cross checked with information provided by the veterinarian at the time of sample submission on the ACDP submission form.

### 2.2. Serological Testing

The HeV serum neutralisation test (SNT) is an accredited test under ISO17025 which is conducted at BSL4 at ACDP. Serial doubling dilutions of sera were carried out in singlicate in a final volume of 50 μL/well. To this, 50 μL of 100 TCID_50_ units of HeV was added and incubated for 30 min at 37 °C. Following incubation, 100 μL Vero cells at 2 × 10^5^-cells/mL was added to each well on the 96 well plate, and the plate was incubated for 3 days at 37 °C, 5% CO_2_ in a humidified incubator. Serum antibody titres are determined as the highest dilution at which there is complete neutralisation of the virus and the absence of cytopathic effect (CPE). Results are captured on reports which were sent to the submitting veterinarian who passed the results onto the owners.

### 2.3. Data Management

Data acquired from Zoetis, the ACDP submission forms and the ACDP testing results were initially entered into Excel spreadsheets which were then migrated to a *PostgreSQL* (v10.16) database. The database structure was normalized to common tables such as owner, horse, vaccinations, veterinarian, and testing results. A PHP website was then constructed to manage and visualise records. All locations recorded within the submission forms were geocoded to the nearest town location using OpenStreetMap’s Nominatim geolocator service available within the GeoPy (v1.20) library using Python 3.7. If a location could not be identified, then the postcode centroid was allocated and the metadata recorded. This spatial data was then used to map owner locations of the vaccinated horses.

Locations of previous Hendra incidents were captured from a Queensland Government website listing all Hendra incidents to date (https://www.business.qld.gov.au/industries/service-industries-professionals/service-industries/veterinary-surgeons/guidelines-hendra/incident-summary; accessed 30 January 2021).

A similar geocoding routine was undertaken to acquire spatial locations of recorded incidents. This allowed the location of Hendra incidents to be mapped combined with the published extents of the *Pteropus alecto* (Black Flying Fox), *Pteropus poliocephalus* (Grey Headed Flying Fox), *Pteropus scapulatus* (Little Red Flying Fox), and *Pteropus conspicillatus* (Spectacled Flying Fox) as identified by the IUCN Red list of Threatened Species [16,17,18,19].

### 2.4. Data and Statistical Analyses

SQL queries were written to obtain summary statistics (median age at sampling, % male and % female, median number of vaccinations per horse, and median time since last vaccination) from the database.

For the statistical analysis, we defined the primary research questions as being how the SNT titres varied with the total number of vaccinations given, as well as the effect of the interval (in days) between the last vaccination and sample collection for SNT. This was on account of this being highly informative as to the vaccination regime i.e., how frequently it needs to be applied to maintain a protective titre. As this question is only relevant following the priming vaccinations, we thus restricted the analysis to the booster vaccinations, i.e., the third vaccination onwards. Furthermore, to minimise the impact of extreme lengths of time between the vaccination and the SNT assay, we restricted the length of time since the last vaccination to be 2 years. This left an analysis dataset of 246 SNT results, on which we applied various exploratory and confirmatory regression analyses.

An exploratory analysis demonstrated the need for a log2 transformation to satisfy the modelling assumption of normally distributed residuals, and upon applying this transformation we compared loess fitted and linear curves, both with and without conditioning on the number of vaccinations. This indicated that a linear model might be suitable in place of a more complex generalised additive model. To assess this, we undertook a formal comparison of statistical models with and without a smoothing term, using both the AIC (Akaike’s Information Criterion) and an Analysis of Deviance for statistical assessment. For this model comparison and the subsequent linear regression modelling, we aggregated the data from vaccination 8, 9, 10, 11, and 12 to overcome the low number of data points for some of these vaccinations.

For the regression modelling undertaken with the aim to find the most parsimonious explanatory variables to predict the SNT response variable, we used a manual backward approach. A full model with the vaccination number, days since vaccination and their interaction was run, and then, explanatory terms were removed based on a non-significant *p*-value (*p* > 0.05).

All statistical analyses were undertaken using *R* release 4.0.2. For the analysis of deviance, we used the “*gam*” functions from the *mgcv* library, with model comparison being done using the “*AIC*” and “*anova*” functions of the *stats* library. For the multiple linear regression, we used the “*lm*” function, also from the *stats* package. All the graphs for the statistical analyses were drawn using the *ggplot2* package.

## 3. Results

### 3.1. Summary Statistics

**Figure 1:**
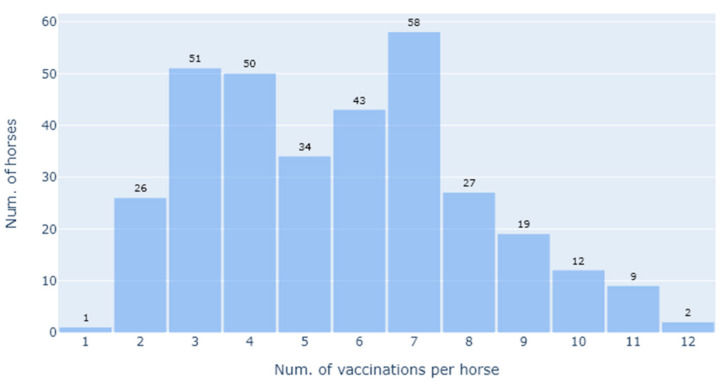
Bar chart showing the number of Equivac^®^ HeV Hendra virus vaccinations per horse at the time of sampling for the SNT, ranging from 1–12, with the count being the number of horses. In total there were data from 332 horses used in this study.

**Figure 2:**
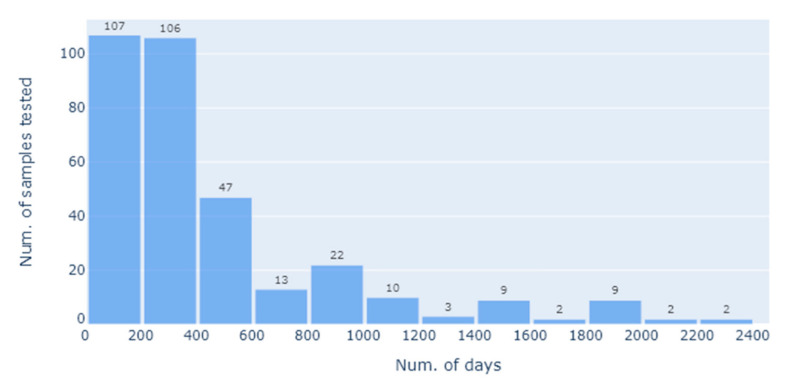
Histogram showing the time between the last Equivac^®^ HeV Hendra virus vaccination and the sampling date for the SNT, with the count being the number of samples tested at that time point (in days); days are grouped for convenience 1–200; 201–400; 401–600, etc.

There were 143 females (43.07%) and 189 males (56.93%) (*n* = 332) used in the data analysis. The median age at sampling was 10 years. The median number of vaccinations per horse was 5 (range 1–12) (Figure 1). Figure 1 is a bar chart showing the number of Equivac^®^ HeV Hendra virus vaccinations per horse with the count being the number of horses. Figure 2 is a histogram showing the time between the last Equivac^®^ HeV Hendra virus vaccination and the sampling date for the SNT, with the count being the number of samples tested at that time point. The time between the last vaccination and sampling extended out to more than 2000 days (>5 years), with most times being less than 400 days (Figure 2).

**Figure 3:**
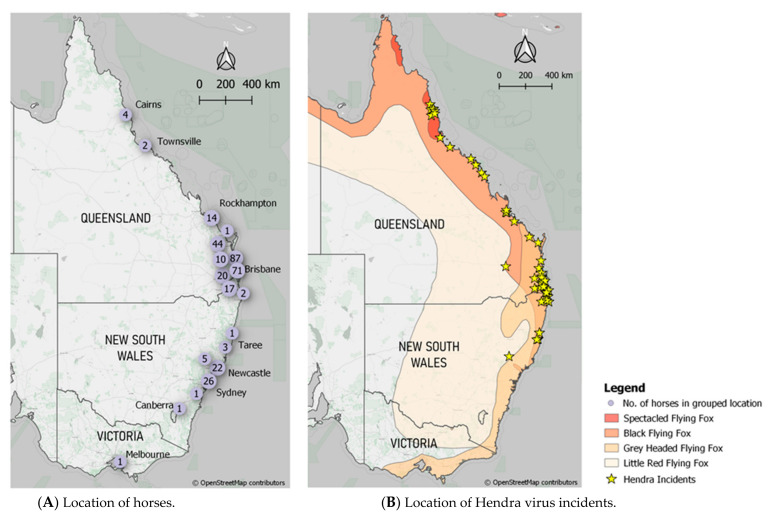
Maps showing (**A**) the location of the Equivac^®^ HeV Hendra virus vaccinated horses in this study and (**B**) the location of the Hendra virus outbreaks with the distribution of the reservoir hosts (*Pteropus alecto*—Black Flying Fox; *Pteropus poliocephalus*—Grey Headed Flying Fox; *Pteropus scapulatus*—Little Red Flying Fox and *Pteropus conspicillatus*—Spectacled Flying Fox) as identified by the IUCN Red list of Threatened Species. Map base tiles were sourced from OpenStreetMap (www.openstreetmap.org/copyright CC BY-SA 2.0) (accessed on 18 June 2021).

The locations of horses in this study are shown in Figure 3A, spreading across three states. This is shown alongside the locations of Hendra virus outbreaks and the distributions of the four flying fox species, the wildlife reservoirs for HeV, on the eastern coast of Australia according to the IUCN (Figure 3B). The majority of vaccinated horses in this study reside in the area where there have been the most Hendra virus outbreaks which is in southeast Queensland and northern New South Wales.

### 3.2. SNT Results

**Figure 4:**
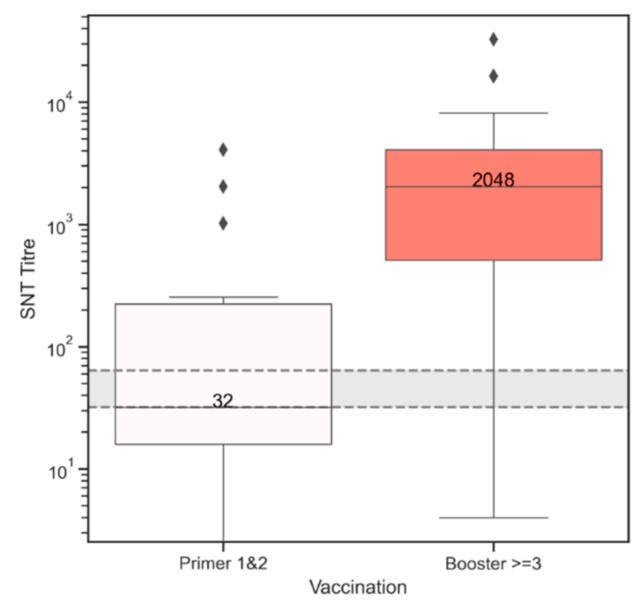
Box and whiskers plot of SNT titre results of horses, split into two groups. The Primer 1&2 group (white) consists of the SNT results from horses which have only received one or two vaccinations, and the Booster ≥3 group (salmon) consists of the SNT results from horses which have received three or more vaccinations. The black diamonds represent outliers above the 95th percentile. Each box shows 25th to 75th percentile; whiskers go between the 5th percentile at the bottom and the 95th percentile at the top. The grey band is between titres 32 and 64 which has been considered a protective titre in previous studies [9].

No horse which had three or more vaccines tested negative. These titres have been presented in a box and whiskers plot, separated by those results from horses in the Primer state (having received only one or two doses) and those in the Booster state (having received doses 3 and above) (Figure 4). The grey band is between titres 32 and 64 which has been considered a protective titre in previous studies [9]. The median titre for Primer 1&2 is 32 (sitting under the hatched line). The median titre for those receiving 3 or more vaccinations is 2048.

In the Primer state, there were three horses which tested negative, but all had only received two vaccines, and the time between last vaccination and sampling date ranged from 1327 to 2393 days, or approximately 3.6 to 6.6 years.

**Figure 5:**
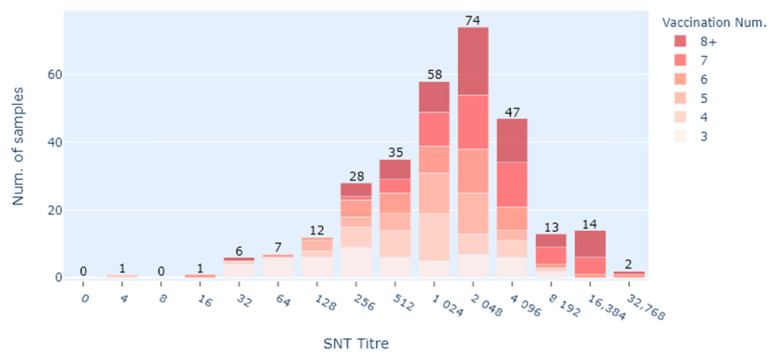
Stacked bar chart showing HeV serum antibody neutralising titres by the number of vaccinations that each horse has had at the time of sampling (3–8+); it does not include those that only had 1 or 2 vaccines (*n* = 298).

When displaying results by the number of vaccinations relative to the antibody neutralising titre, only two horses which had 3 or more vaccinations had titres less than the presumptive protective titre of 32 (Figure 5). The horse which had a titre of 4 had received 3 vaccinations according to the prescribed schedule but had not had the 4th vaccination until 15 months after the 3rd and was sampled 199 days after the last vaccination. The horse which had a titre of 16 had 6 vaccinations and was sampled 198 days after the last vaccination. It is notable that this horse had a 3-year gap between its 3rd and 4th vaccination and a 2-year gap between its 5th and 6th vaccination. The Zoetis recommendation is for annual boosters.

### 3.3. Regression Analysis

**Figure 6:**
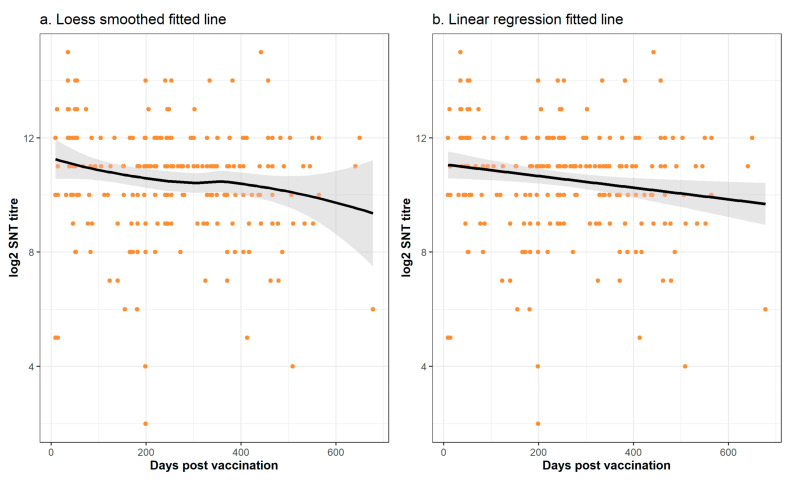
Overall relationship between the time (in days) post-vaccination wherein the serum sample was taken and the measured log2 titre as determined by the serum neutralisation test (SNT) excluding the initial priming regime (vaccinations #1 and 2). Note that whilst the same SNT data are shown in both figures, (**a**) we fit a loess smoothed line whilst in (**b**) we use standard linear regression. For both plots, the grey shading indicates the 95% confidence interval of the fitted lines. There was no significant difference between the loess smoothed and linear regression fitted lines (*p* > 0.05).

The overall relationship between the log2 SNT titre and the days post-vaccination showed a weak decline over time (Figure 6). This applied to both the loess smoothed fitted line as well as to the linear regression fitted line. The comparison of the regression models with and without a smoother showed a slight reduction in the AIC with the smoothed model, but this decrease was not significant (*p* = 0.091). Thus, the more parsimonious linear model was used for the multiple regression analysis.

The full multiple regression model of main and interaction terms showed a highly significant intercept (*p* < 0.001), significance for the higher vaccination numbers as compared to vaccination 3 but no significant effect of the days post vaccination nor any of the interaction terms. However, following model simplification when the interaction terms were removed and the fewer (3 to 6) and the greater number of vaccinations (7 to 12) were each grouped, days post vaccination remains non-significant (*p* = 0.082).

**Figure 7:**
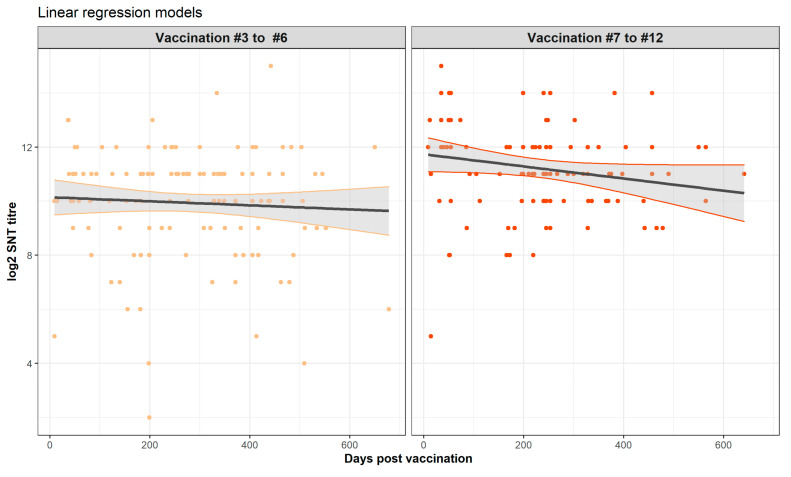
Final regression model showing the fitted line and 95% confidence intervals (grey shading) for vaccination 3 to 6 (left panel) and vaccination 7 to vaccination 12 (right panel) superimposed on the model predicted SNT titres. Note that whilst the intercepts for the two vaccination groupings were highly significantly different (*p* < 0.001), the slopes were not significantly different (*p* > 0.05).

This model simplification results in a final model to explain the log2 SNT titre consisting of only two parameters: the mean value for the fewer vaccination numbers (10.323) and the increase in this mean when 7 or more vaccinations are given (i.e., 10.323 + 1.216 = 11.539). The final model is shown graphically in Figure 7.

## 4. Discussion

In this study, the only commercially available Hendra virus vaccine (Equivac^®^ HeV Hendra virus vaccine) was assessed in its ability to produce neutralising antibody titres. Serological responses in 332 vaccinated horses were evaluated at various times after vaccination. Only three horses did not register a titre, but they had only received two vaccinations, and the time between the last vaccination and sampling was more than 3.5 years. Providing horses had 3 or more vaccinations, neutralising antibody titres were all in the presumed protective range with a median of 2048. There were only two poor responders in this group (3 or more vaccinations), but neither had followed the prescribed vaccination schedule.

Prior to this study, there have been two studies looking at vaccination titres for Hendra virus. In the initial vaccine efficacy study, pre-challenge antibody titres were tested in horses of two groups at 21 days and 194 days after the second HeV vaccination [9]. The lowest antibody titres detected in that study were 16 and 32. All ten vaccinated horses with varied antibody titres (16 to >4096) were protected from lethal exposure to HeV, and no vaccinated horses had a rise in HeV antibodies post exposure [9]. In the second study, HeV vaccination antibody levels were measured over time in a herd of 61 horses that received the primary vaccination course and annual boosters [13]. All horses had an antibody titre of at least 32 after the second vaccination. Vaccination antibody titres were maintained at 16 or above with annual boosters after the primary vaccination course in all but one of the 37 horses remaining in the study [13]. A post vaccination antibody titre of 32 was protective against clinical disease of Nipah virus in a henipavirus vaccine efficacy study in cats which used the same soluble G antigen [20]. Based on these limited data, a protective post vaccination titre of 32 has been previously suggested; however, a cautious approach to recommending a protective post vaccination titre should be taken [15].

Rabies is one virus for which there are widely accepted international standards regarding what is considered a protective post vaccination titre. The WHO recommend that titres of 0.5 IU/mL and above will likely confer protective immunity against rabies virus infection following an exposure [21]. This value has also been adopted by animal health regulators [22]. Despite years of research, the cell-mediated immune response to rabies vaccination has not been thoroughly studied, and gaps in knowledge, especially with respect to longevity of the immune response following rabies vaccination, remain unanswered [23]. Whilst the stimulation of virus neutralising titres is universally considered to be a pre-requisite for protective immunity against rabies infection, the stimulation of memory T-cells is likely to play a role in any anamnestic response following exposure to rabies virus and to long-lived immunity following vaccination [24]. This might be the case with the Equivac^®^ HeV Hendra virus vaccine too; however, it would be difficult to conduct the necessary experiments to prove this due to the containment required with a BSL4 pathogen.

With human rabies vaccinations, annual boosters are not recommended; rather, titres can be checked, and if they fall below the recommended level of 0.5 IU/mL, then a booster is warranted. Rabies virus-specific neutralising antibodies have been shown to be detectable in humans for up to 14 years after vaccination [25]. Despite this recommendation for humans, the rabies vaccination schedule for animals includes annual boosters.

The Hendra vaccination schedule originally had six-monthly boosters until data from Tan et al. [13] were submitted to the AVPMA, supporting the change to annual boosters which came into effect in 2016. The Hendra vaccination schedule is now no more arduous than the schedules for the most commonly administered equine vaccines in Australia which are those for tetanus and strangles (R. Le Strange pers com.). These have a similar priming schedule: the tetanus vaccine has two doses administered 2–4 weeks apart; the strangles vaccine has three doses administered 2 weeks apart. Both have annual boosters which can be conveniently administered as a 2-in-1. During Australia’s equine influenza (EI) outbreak in 2007, the recommended vaccination schedule for the recombinant canarypox EI vaccine which was used to control the outbreak had a primary course (first immunisation (V1) from 5 to 6 months of age with a second immunisation (V2) 4–6 weeks later) and revaccination 5 months later (V3) [26]. Antibody responses to EI vaccines are well documented. Establishment of protective levels of serial radial haemolysis (SRH) antibodies is considered sufficient in some cases for registration of equine influenza vaccines in Europe, and antibody decline has been extensively followed in individuals [27,28]. A limitation of the current study was that it largely consisted of serum samples collected from horses at a single point in time. There would be value in following the responses of individual horses over a period of time, to better understand the duration of immunity and antibody decline in individuals.

Another limitation of this study was that it used results from the SNT which is a biological assay, and by virtue of this, it is inherently imprecise. Advice on interpretation involves +/− one dilution, meaning a titre of 64 could be as low as 32, or as high as 128. Assessing antibody responses using different methodologies, such as the Luminex binding assay, might provide more refined data and clarity around the pattern of responses [29].

These two study limitations, *viz*., that the sampling was not structured and the biological imprecision of the SNT assay, can explain the apparent inconsistency from the formal statistical analyses of a lack of significant trend between vaccination and the sampling time. From comparison of this relationship from other vaccines, we might have expected a non-linear relationship with an initial rising titre which gradually falls over time. Whilst this is suggested by visual inspection of the graphs of the time trends (Figure 6 and Figure 7), this was not confirmed by the formal statistical analysis which concluded that the non-linear relationships and trends were not significant. Nevertheless, it is important to note that regression model building is based on the goodness of fit of a test statistic to the data, and this can be highly sensitive to non-normality, outliers, and high data variability [30]. To overcome the first two of these challenges to statistical model building, we used a log transformation and exclusion of the data points sampled beyond 2 years. However, the high variability of the data was an intrinsic part of the study and cannot be reduced in a statistically defendable manner. Thus, whether there is a linear or a curvilinear relationship between the sampling time and the SNT titre cannot be definitively answered by this study and requires a sampling and testing experimental design with less inherent variability.

Such a design to determine the true biological trend of the SNT titre over the time following vaccination might best be achieved through a longitudinal study where individual horses are sampled before each vaccination and then at fixed time points following this, with the antibody response determined with a less variable assay (see above). Nevertheless, this would be a lengthy study and whilst scientifically interesting, it is debatable whether this is truly the best follow-on approach. Currently in Australia there is a degree of “vaccine hesitancy” by horse owners to use the HeV vaccine with the frequency of boosters being one of the identified influencing factors [12]. The finding from our study that protective titres can last up to 24 months indicates a biennial booster vaccination regime is possible, which might also reduce this vaccine hesitancy. Nevertheless, the rigour of Australia’s vaccine regulatory procedures will require that there be strong supporting evidence, which in turn might require a formal double-blind, randomised clinical trial in a susceptible population.

## 5. Conclusions

In conclusion, this study shows that the Equivac^®^ HeV Hendra virus vaccine induces significant neutralising antibody titres following the completion of the priming schedule and annual boosters. Furthermore, if horses receive seven or more annual boosters, they are likely to have even higher titres. However, further research is needed to evaluate neutralising antibody decline.

## Data Availability

Blinded SNT data are available from the corresponding author upon reasonable request. Sample metadata are not available due to privacy restrictions afforded to diagnostic submissions.

## Supplementary Materials

The following are available online at https://www.mdpi.com/article/10.3390/vaccines9070731/s1, Table S1. HeV vaccine doses sold; Table S2. Estimated horse population in Australia and percentage of horses HeV vaccinated.
