# Supplementary material for: Sero-Monitoring of Horses Demonstrates the Equivac® HeV Hendra Virus Vaccine to Be Highly Effective in Inducing Neutralising Antibody Titres
Source: Vaccines (Basel). 2021 Jul 2;9(7):731. doi: 10.3390/vaccines9070731 (PMC8310234; doi:10.3390/vaccines9070731)
Supplement: Supplementary file 1 [file vaccines-09-00731-s001.zip › vaccines-1230526-supplementary.pdf]

Supplementary Information

Table S1. HeV vaccine doses sold*

Number of vaccine doses sold in each calendar year from 2016 to 2019. The left column is the total number of doses sold in each year. The middle column is the total number of individual horses that received at least one dose in that year (some will have received more than one dose but are only counted once). The right column is the number of horses receiving their first ever dose in that year. The spike in numbers in 2019 corresponds with the outbreak in Scone.

Year	Number of doses sold	Total number of horses receiving at least one dose	Number of horses receiving first dose
2016	76,056	55,471	12,566
2017	73,496	55,000	12,233
2018	70,400	53,211	10,243
2019	97,078	66,285	22,603
**TOTALS**	**317,030**		**57,645**

*Data provided by Zoetis.

Table S2. Estimated horse population in Australia and percentage of horses HeV vaccinated

Estimating the percentage of horses in Australia receiving at least one dose of the vaccine in one year, based on horse population estimates [14] and data from S1 Table of HeV vaccine doses sold. In the endemic states, vaccination coverage between 2016 and 2019 ranged from 10.1% to 13%, which at a national level decreases to 6.9% to 8.9%. This is low compared to other vaccines such as tetanus where approximately twice as many doses are sold per year (R. Le Strange pers. com).

Year	Est. horse population in Australia	Est. horse population in QLD & NSW	Est. % horses HeV vaccinated in Australia	Est. % horses HeV vaccinated in QLD & NSW
2016	799,000	547,400	6.9%	10.1%
2017	781,200	534,600	7.0%	10.2%
2018	763,400	521,800	7.0%	10.2%

2019	745,600	509,000	8.9%	13.0%
